# Microbiome analysis reveals gut microbiota alteration of early-weaned Yimeng black goats with the effect of milk replacer and age

**DOI:** 10.1186/s12934-021-01568-5

**Published:** 2021-03-31

**Authors:** Aoyun Li, Yan Yang, Songkang Qin, Shenjin Lv, Taihua Jin, Kun Li, Zhaoqing Han, Yongzhu Li

**Affiliations:** 1 College of Agriculture and Forestry Science, Linyi University, Linyi, China; 2 College of Veterinary Medicine, Huazhong Agricultural University, Wuhan, 430070 China; 3 Institute of Traditional Chinese Veterinary Medicine, College of Veterinary Medicine, Nanjing Agricultural University, Nanjing, 210095 China; 4 Linyi Academy of Agricultural Sciences, Linyi, 276012 China

**Keywords:** Milk replacer, Gut microbiota, Yimeng black goats, Weaned, Age

## Abstract

**Background:**

Colonization of intestinal microbiota in ruminant during the early life is important to host health, metabolism and immunity. Accumulating evidence revealed the ameliorative effect of milk replacer administration in the gut microbial development of early-weaned ruminants. Yimeng black goats (YBGs) inhabiting Shandong, China show a complex intestinal microbial ecosystem, but studies of their gut microbiota are still insufficient to report. Here, this study was performed to investigate how the gut microbiota develops in weaned YBGs with the effect of age and milk replacer.

**Results:**

Results indicated that both age and milk replacer were important factors to change the gut microbiota of YBGs. Although the alpha diversity of gut microbiota did not change with the age of YBGs, the taxonomic compositions significantly changed. The relative abundance of some beneficial bacteria such as *Lachnospiraceae*, *Ruminococcaceae*, *Ruminiclostridium*, *Eubacterium* and *Barnesiella* significantly decreased and subsequently increase with age, which contributes to maintain the stability of intestinal environment and realize the diversity of intestinal functions. The relative abundance of *Porphyromonas*, *Brevundimonas*, *Flavobacterium*, *Stenotrophomonas*, *Propionibacterium*, *Acinetobacter*, *Enterococcus* and *Clostridium* belong to pathogenic bacteria in milk replacer-treated YBGs was significantly decreased. Additionally, some beneficial bacteria such as *Ruminococcus*, *Ruminococcaceae*, *Christensenellaceae* and *Ruminiclostridium* also display a trend of decreasing first followed by gradually increasing.

**Conclusions:**

This study first revealed the gut bacterial community alterations in YBGs with the effect of age and milk replacer. This study also characterized the gut microbial distribution in YBGs with different ages and provided better insight into microbial population structure and diversity of YBGs. Moreover, milk replacer may serve as a good applicant for improving gut microbial development in early-weaned YBGs.

## Introduction

Ruminant gut microbiota composed of trillions of microorganism is the most complicated and largest micro-ecosystem, which plays vital roles in mucosal immunity, nutrient absorption and intestinal epithelium differentiation [1, 2]. Furthermore, the gut microbiota may also serves as a contributing or central factor of various diseases, affecting both near and far organ systems [3]. Generally, these microorganisms inhabiting in gut such as bacteria, fungi and archaea can interact in a commensal, symbiotic or parasitic relationship resulting in stabilizing and maintaining the intestinal environment [4, 5]. The stabilized gut bacterial community is the precondition for host performing the normal physiological functions, metabolism and immune function, but gut microbiota imbalance may result in multiple gastrointestinal diseases, such as diarrhea and irritable bowel syndrome [6, 7]. Ruminants displayed unique digestive properties and microbial population that enables them to evolve special adaptations in high fiber content foods, but also make them susceptible to multiple gastrointestinal diseases [8]. Given the importance of ruminant gut microbiota in many physiological functions, investigating the development and composition of its microbial community is of great significance.

Milk replacer is the artificial milk produced by replacing milk protein with non-milk protein on the basis of the nutritional standards of breast milk [9]. The nutritional components and physical form of milk replacer are similar to the breast milk, and its quality is not easily affected by the external environment [10]. Several evidence demonstrated that milk replacer administration during the ruminant juvenile period could improve the immunity and decrease the stress response caused by the sudden changes of feed morphology [11]. Moreover, milk replacer administration could also ameliorate the growth performance of kids and reduce the morbidity and mortality caused by insufficient early nutritional intake [12]. Remarkably, several recent studies indicated the beneficial effects of milk replacer administration in the early-weaning calves and kids for regulating their gut microbiota [13]. Yimeng black goats (YBGs) are an indigenous breed of the Shandong, China characterized by outstanding adaptability and stress resistance [13–15]. However, the quantity of YBGs is relatively small because of low fertility rate and delayed growth. Our previous research has demonstrated the ameliorative effect of milk replacers on the growth performance and rumen microbiota of YBGs [14]. However, studies regarding the influence of milk replacer on gut microbiota in YBGs have been insufficient to date. Taking advantage of this gap, we investigated the variability of gut microbiota in the YBGs with the effect of age and milk replacer.

## Materials and methods

### Animals and sample acquisition

A group of 24 one-day-old healthy YBGs with similar initial weight were obtained from a commercial feedlot (Shandong, China). The YBGs purchased for this experiment were self-propagated by the commercial feedlot and had similar genetic backgrounds. All the YBGs were randomly divided into control group (B group) and milk replacer administration group (R group). The YBGs were raised in experimental animal center, Linyi University (Shandong, China) for 75 days and provided with the recommended clean environment and breeding temperature. The control and experimental groups were compulsively weaned on day 10, but the experimental group was provided with milk replacer after weaning. Moreover, adequate starter feed and water were provided ad libitum from day 15. The nutrient composition of milk replacer and starter feed was based on our previous research [14]. Three YBGs from each group were randomly selected to sacrifice for obtaining the intestinal samples on days 15, 25, 45 and 75. All the YBGs were euthanized by injecting pentobarbital (25 mg/kg). After euthanizing, the intestines were stripped from the mesentery by using sterilized surgical knife. The intestines (duodenal, ileum, jejunal and cecum) were knotted using cotton ropes to minimize the potential cross-contamination among the different intestines. After that, the contents from the intermediate areas of the different intestines were carefully collected. The collected intestinal samples were immediately stored in the sterilized tubes, snap-frozen using liquid nitrogen and stored at − 80 ℃ for further study.

### DNA extraction and illumina MiSeq sequencing

Intestinal content samples were subjected to bacterial genomic DNA extraction via using QIAamp DNA Mini Kit (QIAGEN, Hilden, Germany) according to the suggested instructions of manufacturer. The integrity and size of collected DNA was verified by 0.8% agarose gel electrophoresis and the UV–Vis spectrophotometer (NanoDrop 2000, United States) was used for determining the DNA concentrations. The primers (338F: ACT CCT ACG GGA GGC AGC A and 806R: GGA CTA CHV GGG TWT CTA AT) with adaptors, which were designed according to the conserved region, were used for amplifying the V3/V4 regions. PCR amplification was performed in triplicated as described previously. The evaluation and purification of PCR amplification products were performed by using 2.0% agarose gel electrophoresis and AxyPrep DNA Gel Extraction Kit (Axygen, CA, USA), respectively. Purified PCR products were fluorescently quantified on Microplate reader (BioTek, FLx800) on the basis of the initial quantitative results of electrophoresis. Subsequently, each sample was mixed in corresponding proportion based on the fluorescence quantitative results and the sequencing quality requirements. The purified PCR amplification products were used for generating the sequencing library using Illumina TruSeq (Illumina, United States) according to manufacturer’s specifications. The sequencing libraries were conducted quality inspection and fluorescence quantification prior to sequencing. The qualified libraries shown only a single peak and the concentration is more than 2 nM. The collected libraries were assembled, diluted and mixed in proportion based on the quantity of sequencing required. Finally, the libraries were performed high-throughput sequencing by using MiSeq sequencing machine.

### Bioinformatics and data analysis

The paired-end sequences obtained from sequencing were merged into a tag and the quality of raw reads were screened. Specifically, the reads of each sample were spliced through overlap using FLASH software (v1.2.7) to obtain original tags. Moreover, the Trimmomatic (v0.33) software and UCHIME (v4.2) software were used to filter the original tags and eliminated chimera, respectively to achieve effective Tags. The obtained high-quality sequences were clustered to the same OTU on the basis of 97% similarity. Representative sequence of each OTU was performed classification identification and phylogenetic analysis. The alpha diversity was calculated based on the abundance distribution of OTUs in different samples. Beta diversity was performed using QIIME (Version 1.7.0) to compare the difference and similarity among different samples. Moreover, the rarefaction curves were constituted to assess the sequencing depth. Linear discriminant analysis effect size (LEfSe) was generated to analyse the differentially abundant taxon. R (v3.0.3) and GraphPad Prism (version 7.0c) were applied to statistical analysis. P-values < 0.05 were considered statistically significant and the data was expressed as means ± SD.

## Results

### Sequences analyses

**Fig. 1:**
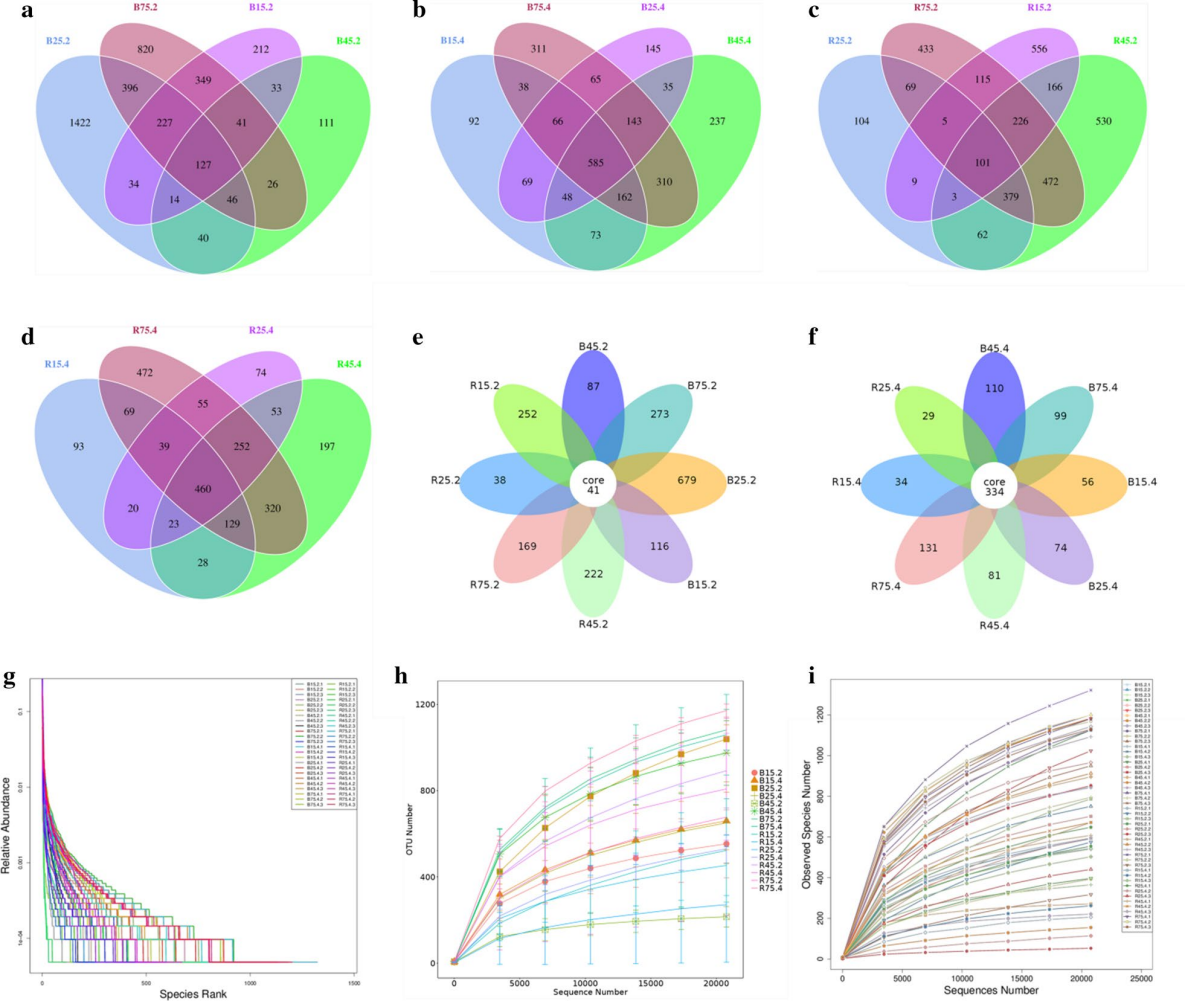
Operational taxonomic units (OTUs) and sample feasibility analysis. Venn diagrams show unique and shared bacterial OTUs in jejunum (**a**) the jejunum in control group; (**c**) the jejunum in milk replacer-treated group) and cecum (**b**) the cecum in control group, (**d**) the cecum in milk replacer-treated group). Venn diagrams for core OTUs distribution in all the jejunum (**e**) and cecum (**f**). Rank-Abundance (**g**) and rarefaction curves (**h**, **i**) were used for the assessment of sequencing depth. Each color-curve represents a sample. B15.2, B25.2, B45.2 and B75.2 represent the jejunum in the control group on the days 15, 25, 45 and 75, respectively. B15.4, B25.4, B45.4 and B75.4 represent the cecum in the control group on the days 15, 25, 45 and 75, respectively. R15.2, R25.2, R45.2 and R75.2 indicate the jejunum in the milk replacer-treated group on the days 15, 25, 45 and 75, respectively. R15.4, R25.4, R45.4 and R75.4 indicate the cecum in the milk replacer-treated group on the days 15, 25, 45 and 75, respectively

Following taxonomic assignment, a total of 11,789 OTUs were recognized based on 97% nucleotide-sequence similarity with an average of 245 OTUs per sample. On average, 3898, 2379, 3228 and 2284 OTUs belonged to control jejunum, control cecum, milk replacer treated jejunum, milk replacer treated cecum, respectively (Fig. 1a–d). Moreover, we also observed 41 core OTUs in all the jejunum as well as 334 core OTUs were identified in all the cecum (Fig. 1e, f). The rank abundance curve of each sample is wide and falls gently, displaying the excellent evenness and richness (Fig. 1g). Moreover, both OTU curve and species accumulation curve were relatively flat and displayed a tendency to saturate as the number of qualified sequences exceed 20,000, indicating that the sequencing quantity and depth meet the requirement for sequencing and analysis (Fig. 1h, i).

### Alterations in gut microbial diversities with the effect of milk replacer and age

**Fig. 2:**
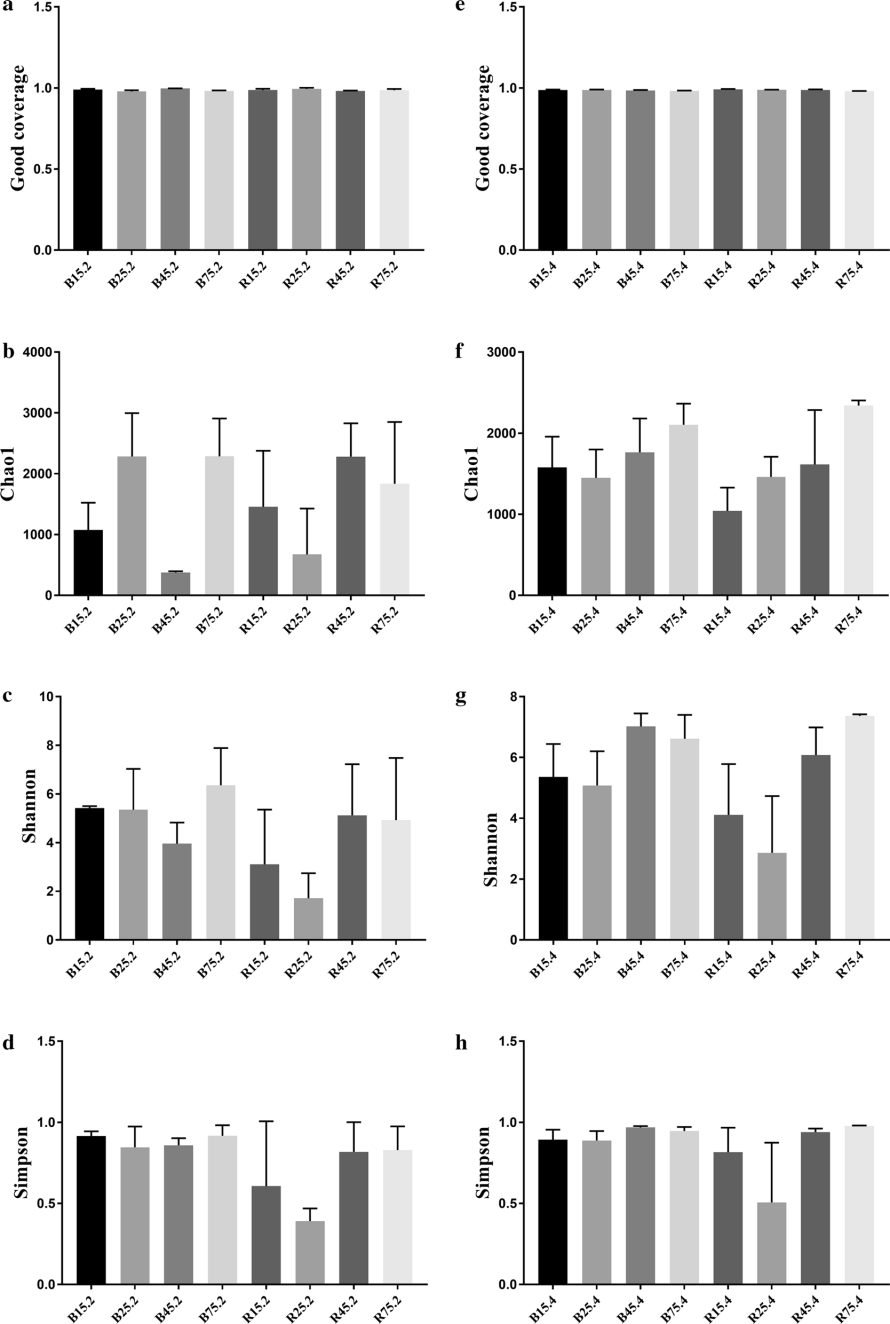
Microbial diversity index analysis. Good’s coverage (**a**, **e**), Chao1 (**b**, **f**), Shannon (**c**, **g**) and Simpson (**d**, **h**) were applied to assess the alpha diversity of gut microbial community

To assess the differences in the diversity and abundance of gut microbiota between different groups the qualified sequences achieved in the sequencing were aligned to estimate alpha index. Alpha diversity of gut microbial population could be reflected by community abundance (Chao1), diversity index (Shannon and Simpson) and sequencing depth (Good's coverage). Good’s coverage estimates in all the groups were approximately 100%, implying the excellent coverage (Fig. 2a, e). We observed that Chao1, Simpson and Shannon indices did not change significantly with age, implying that the diversity and abundance in intestinal microbiota of YBGs did not change from days 15 to 75 (Fig. 2b–d). Moreover, there was no statistically distinct difference in three diversity indices between both groups, indicating that milk replacer administration had no effect on the abundance and diversity of the intestinal microbiota of YBGs (Fig. 2f–h).

**Fig. 3:**
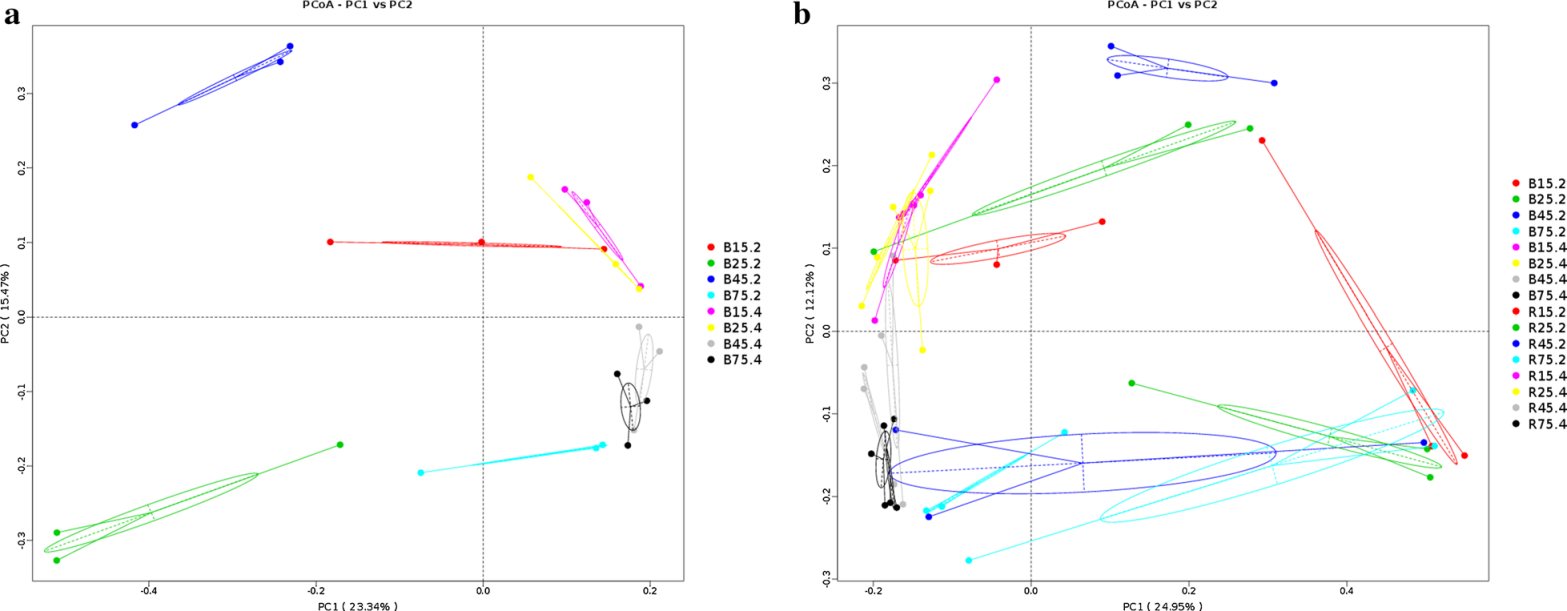
Differences in principal component of gut microbial structures. Each colored point in the figure represents one sample. The distance between the two points indicates the difference of gut microbiota

The PCoA plots based on the weighted UniFrac distances indicated that despite of shared diets and growing environment, the YBGs displayed continuous alterations in their gut microbial communities with age (Fig. 3a). However, the individuals in same group were clustered together, indicating that the intestinal microbiota composition between the samples in one group was similar. PCoA plots also revealed a separation of the jejunum and cecum, suggesting that the principal compositions of gut microbiota between jejunum and cecum were significantly different. Moreover, the samples in both groups gradually clustered with time, indicating that milk replacer administration had no effect on the main compositions of gut microbiota (Fig. 3b).

### Significant alterations in the gut microbial compositions with the age of YBGs

**Fig. 4:**
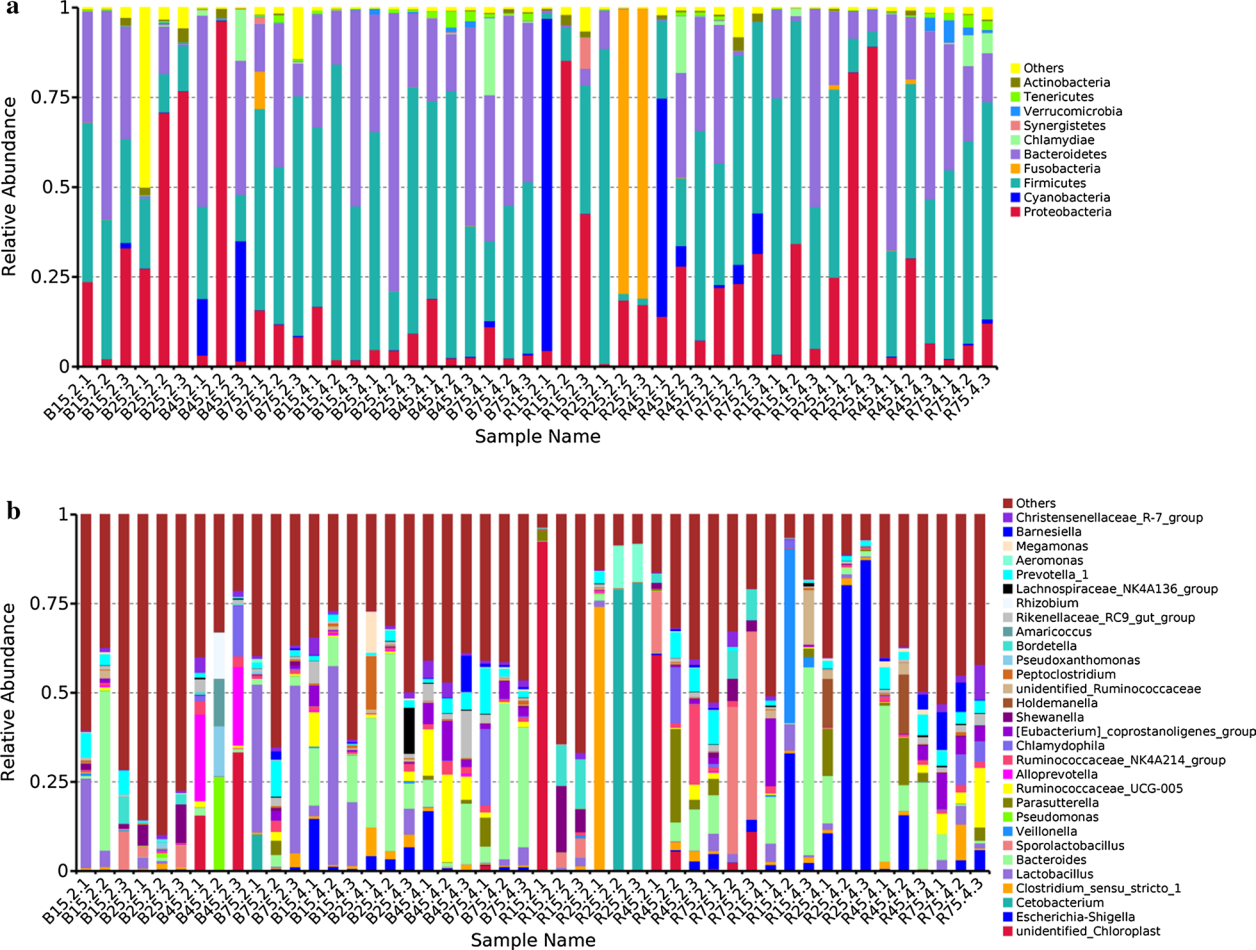
Taxonomic distribution of different samples at the levels of phylum (**a** top ten) and genus (**b** top thirty). Each color-block represents the relative abundance of a bacterial taxon in a sample

**Fig. 5:**
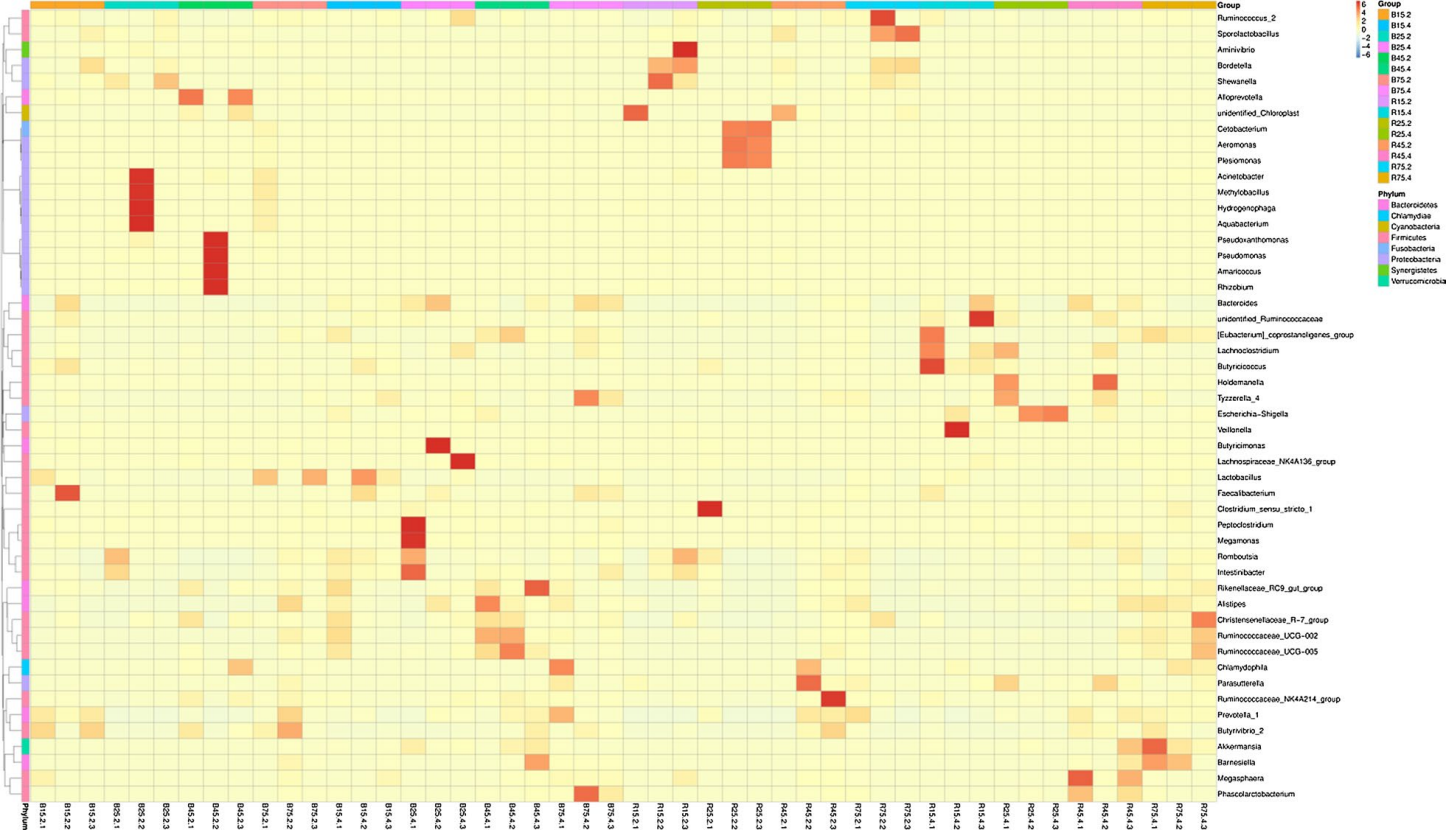
Heatmap displays the top 50 most preponderant bacterial genera in control and milk replacer treated YBGs. The relative richness of each genus is indicated by color intensity. The blue to red gradient indicates the alteration in abundance from low to high

The relative proportion of preponderant taxa at the levels of phylum and genus were assessed through microbial taxa assignment in different groups (Fig. 4). According to the phylum assignment result, phyla *Firmicutes*, *Proteobacteria* and *Bacteroidetes* were the most dominant bacteria in the control YBGs regardless of age, accounting for approximately 90% of the taxonomic groups identified. Additionally, bacteria belonging to the phyla *Synergistetes*, *Verrucomicrobia*, *Tenericutes* and *Actinobacteria* were represented with a lower abundance in all the samples. At the genus level, *Bacteroides* (15.28%), *Lactobacillus* (9.95%) and *Prevotella_1* (5.61%) were the three most dominant genera in the jejunum of 15-day-old YBGs, whereas *Shewanella* (5.54%), *Sporolactobacillus* (3.02%) and *Lactobacillus* (1.33%) were observed as the predominant in the jejunum of 25-day-old YBGs. Moreover, the most abundant genera were *unidentified_Chloroplast* (16.40%), *Alloprevotella* (15.48%) and *Pseudomonas* (8.76%) in the jejunum of 45-day-old YBGs, while the occurrence of *Lactobacillus* (29.62%), *Prevotella_1* (58.13%) and *Cetobacterium* (4.48%) was higher in the jejunum of 75-day-old YBGs. *Lactobacillus* (25.58%) and *Ruminococcaceae_UCG-005* (14.73%) were the most predominant bacterium in the cecum of 15-day-old and 45-day-old YBGs, followed by *Bacteroides* (12.45%, 8.58%) and *Escherichia-Shigella* (5.64%, 6.05%), which together made up 43.67% and 29.36% of the overall bacterial composition, respectively. Moreover, *Bacteroides* (30.99%), *Peptoclostridium* (5.14%) and *Escherichia-Shigella* (4.86%) were the most prevalent bacteria in the cecum of 25-day-old YBGs, whereas *Bacteroides* (27.30%), *Chlamydophila* (7.33%) and *Prevotella_1* (5.55%) were observed predominant in the cecum of 75-day-old YBGs. Moreover, The distribution of bacterial genera in each sample can also be observed in the heatmap (Fig. 5).

**Fig. 6:**
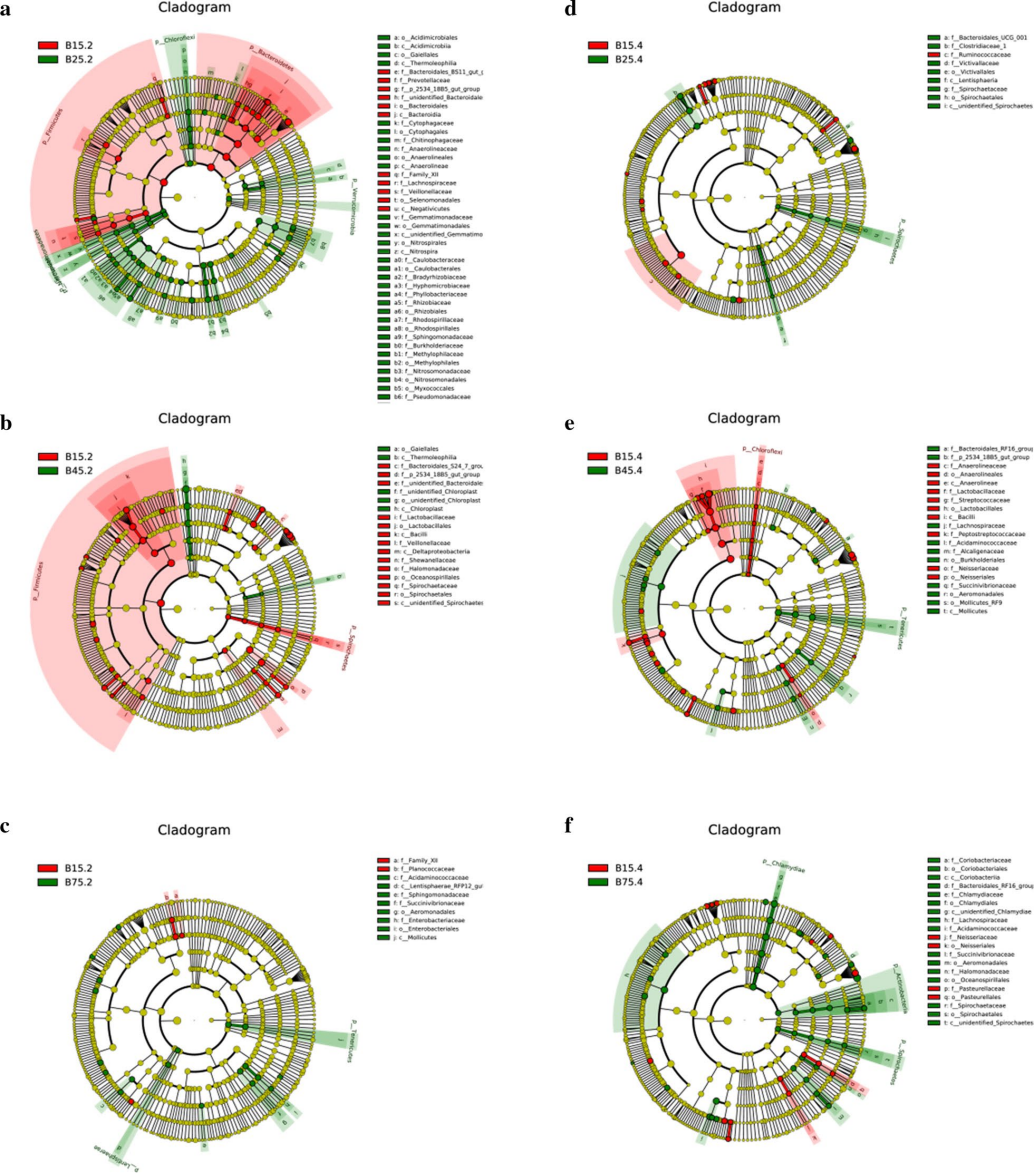
Cladogram obtained from LEfSe analysis shows the different taxa in microbiota of YBGs with different ages. The colored circles from the inside to the outside represent the taxonomic levels of phylum, class, order, family, and genus. The yellow circles in the cladogram indicate the taxa with no significant differences

Using LEfSe analysis to compare the bacterial genus-level taxonomic compositions among groups, we found that the relative abundances of *Pseudobutyrivibrio*, *Eubacterium_ventriosum_group* and *Eubacterium_nodatum_group* in the B75.2 were higher than that in the B15.2. However, the relative abundances of *Prevotellaceae_UCG_003* and *Prevotella_1* in the B25.2, and *Ruminococcus_2*, *Clostridium_sensu_stricto_1* and *Butyricicoccus* in the B45.2 was lower than that in the B15.2, respectively (Fig. 6). The relative abundances of *Clostridium_sensu_stricto_1* in the B25.4, *Pseudobutyrivibrio*, *Lachnospiraceae_NK3A20_group*, *Lachnospiraceae_UCG_001*, *Lachnospiraceae_AC2044_group* and *Bifidobacterium* in the B45.4, *Lachnospiraceae_XPB1014_group*, *Prevotellaceae_UCG_003*, *Prevotellaceae_UCG_001*, *Ruminococcus_1*, *Lachnospiraceae_AC2044_group*, *Lachnospiraceae_ND3007_group* and *Lachnospiraceae_UCG_001* in the B75.4 were significantly higher than that in the B15.4, respectively, whereas the relative abundances of *Ruminiclostridium_9* in B25.4, and *Ruminiclostridium* in B45.4 were lower. Moreover, some pathogenic bacteria such as *Moraxella*, *Streptococcus* and *Turicibacter* in the B15.4 were significantly higher than that in the B45.4. Interestingly, the relative abundances of *Lachnospiraceae*, *Ruminococcaceae* and *Ruminiclostridium* in jejunum and *Ruminococcaceae*, *Eubacterium_ruminantium_group* and *Barnesiella* in cecum significantly decreased and subsequently increased.

### Significant alterations in the gut microbial compositions of YBGs with the effect of milk replacer

The phylum *Proteobacteria* was the most preponderant bacteria in the jejunum of 15-day-old YBGs treated by milk replacer, followed by the phyla *Cyanobacteria* and *Firmicutes* (Fig. 4). Moreover, the phylum *Fusobacteria* was much more abundant in the jejunum of 25-day-old YBGs treated by milk replacer than other phyla, whereas the phyla *Firmicutes* and *Proteobacteria* were the second and third most abundant, respectively. In contrast, the predominant bacteria in the jejunum of 45-day-old YBGs treated by milk replacer was the phylum *Firmicutes*, followed by the phyla *Cyanobacteria* and *Bacteroidetes*, which accounted for over 75% of all bacterial taxa. Additionally, the dominant phyla found in jejunum of milk replacer administration 75-day-old YGBs were also *Proteobacteria*, *Firmicutes* and *Bacteroidetes*, which were consistent with results in the control group. In the cecum of milk replacer administration YGBs, *Proteobacteria*, *Firmicutes* and *Bacteroidetes* were the three most preponderant phyla, which accounted for more than 93% of the total 16S rRNA gene sequences. At the genus level, *unidentified_Chloroplast* (30.83%) was the most dominant bacterium in the jejunum of 15-day-old YGBs treated by milk replacer, followed by *Bordetella* (8.51%) and *Shewanella* (8.31%), which together made up 47.65% of the bacterial composition. *Cetobacterium* (53.26%), *Clostridium_sensu_stricto_1* (24.75%) and *Aeromonas* (7.59%) were the most predominant bacteria in the jejunum of 25-day-old YGBs treated by milk replacer, while *unidentified_Chloroplast* (22.12%), *Parasutterella* (9.73%) and *Ruminococcaceae_NK4A214_group* (7.94%) were observed to be predominant in the jejunum of 45-day-old YGBs treated by milk replacer. *Sporolactobacillus* (31.33%) was the prevalent bacteria in the jejunum of 75-day-old YGBs treated by milk replacer, followed by *Bordetella* (5.49%) and *unidentified_Chloroplast* (4.77%). *Bacteroides* (22.00%), *Veillonella* (17.50%) and *Escherichia-Shigella* (12.49%) were abundantly present in the cecum of 15-day-old YGBs treated by milk replacer, whereas the occurrence of *Escherichia-Shigella* (59.47%), *Bacteroides* (5.51%) and *Parasutterella* (4.63%) were high in the cecum of 25-day-old YGBs treated by milk replacer. *Bacteroides* (25.09%), *Holdemanella* (5.66%) and *Parasutterella* (5.57%) were the most prevalent bacteria in the cecum of 45-day-old YGBs treated by milk replacer, while *Ruminococcaceae_UCG-005* (8.56%), *Eubacterium_coprostanoligenes_group* (6.40%) and *Barnesiella* (6.36%) were the most abundant bacteria in the cecum of 75-day-old YGBs treated by milk replacer.

**Fig. 7:**
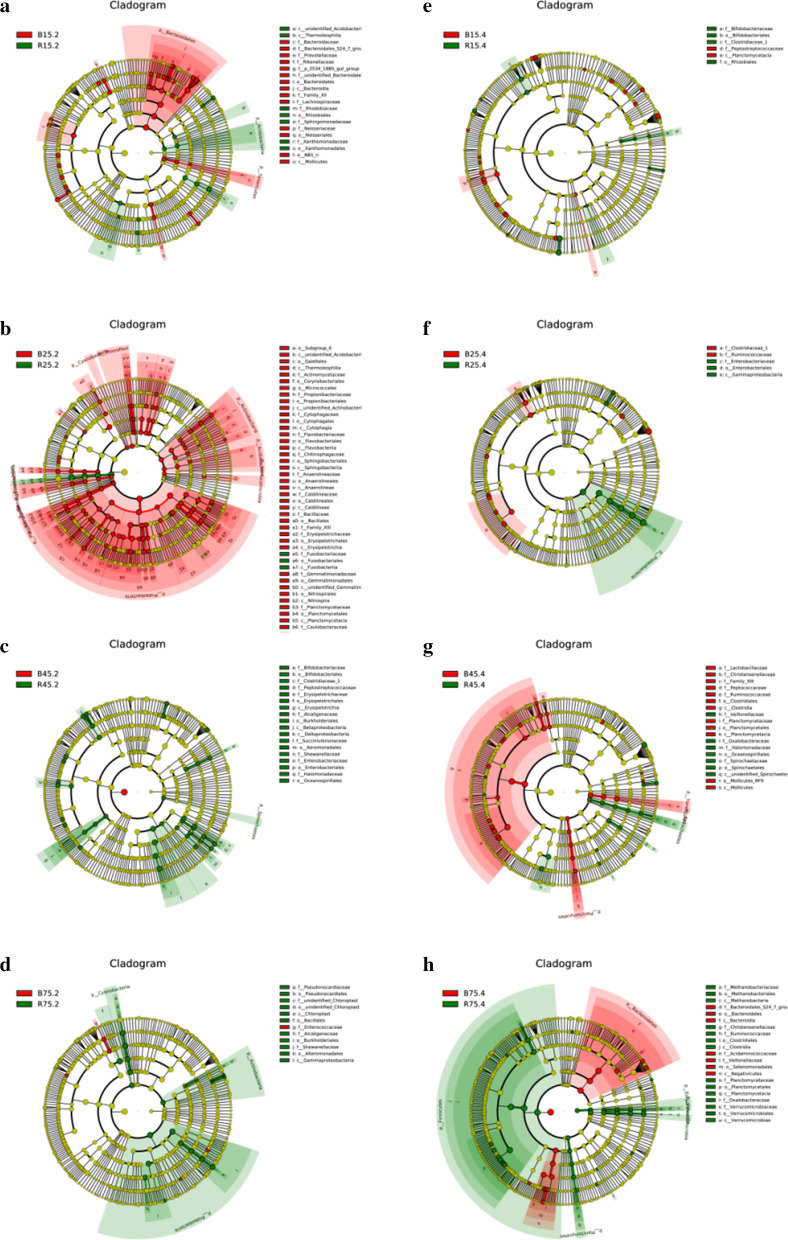
LEfSe analysis indicated the differences in the relative abundance of control and milk replacer-treated groups. Cladogram reveals the phylogenetic distribution of gut bacterial community associated with control and milk replacer-treated groups. The taxa with no distinct differences was represented by the yellow circles

LEfSe analysis was performed to check the significant difference between control and milk replacer administration YBGs segments on the basis of taxa (phylum to genus) identification (Fig. 7). At the phylum level, the abundance of the *Cyanobacteria*, *Actinobacteria* and *Proteobacteria* were significantly decreased in R25.2 in comparison with B25.2. Additionally, *Firmicutes* was dramatically abundant in R75.4 than in the B75.4, whereas the abundance of *Bacteroidetes* was lower. At the genus level, *Micrococcus* level tended to be higher in the R15.2 than B15.2, whereas the *Prevotellaceae_NK3B31_group*, *Prevotellaceae_UCG_001*, *Ruminococcus_1*, *Ruminiclostridium*, *Ruminococcaceae_UCG_005*, *Prevotellaceae_UCG_003*, *Porphyromonas* and *Prevotella_1* displayed the opposite trend. The relative abundance of *Pseudobutyrivibrio*, *Ruminococcaceae_UCG_014*, *Brevundimonas*, *Flavobacterium*, *Turicibacter*, *Stenotrophomonas*, *Propionibacterium*, *Halomonas* and *Acinetobacter* in the R25.2 were significantly lower than the B25.2. Meanwhile, the R45.2 was significantly enriched for *Lachnospiraceae_NK3A20_group*, *Ruminococcus_2*, *Ruminococcaceae_UCG_014*, *Bifidobacterium* and *Lachnospiraceae_XPB1014_group* in comparison with B45.2. Moreover, a comparison of the R75.2 and B75.2 displayed a significant increase in the abundance of *Shewanella* as well as a obvious decrease in the abundance of *Acinetobacter* and *Enterococcus*. The abundance of *Clostridium_sensu_stricto_1* in the R15.4 was found relatively higher, whereas the levels of *Tyzzerella_3*, *Eubacterium_ruminantium_group*, *Ruminococcaceae_UCG_014*, *Ruminococcaceae_UCG_013* and *Ruminiclostridium_9* were lower as compared to B15.4.

The comparison of these identified taxa indicated that the B25.4 had a significantly higher abundance of *Ruminiclostridium*, *Lachnospiraceae_FE2018_group*, *Lachnospiraceae_UCG_010*, *Ruminococcaceae_UCG_005*, *Clostridium_sensu_stricto_1* and *Butyricimonas* than those of R25.4, while the relative abundance of *Prevotellaceae_NK3B31_group* was lower. *Lachnospiraceae_UCG_010*, *Ruminiclostridium_9*, *Ruminococcaceae_UCG_009*, *Ruminiclostridium_5*, *Prevotellaceae_UCG_004*, *Ruminococcaceae_NK4A214_group*, *Christensenellaceae_R_7_group* and *Ruminococcaceae_UCG_005* levels tended to be higher in B45.4 than R45.4, while the *Roseburia* and *unidentified_Lachnospiraceae* showed the opposite trend. Moreover, R75.4 were characterized by high levels of *Ruminococcaceae_UCG_005*, *Christensenellaceae_R_7_group*, *Akkermansia*, *Ruminococcaceae_UCG_002*, *Ruminococcaceae_UCG_010*, *Ruminococcaceae_UCG_013*, *Ruminiclostridium_5* and *Ruminococcaceae_UCG_009* compared with B75.4.

## Discussion

Ruminant gut microbiota is an interactive and complex system, which play key roles in metabolism, immunity, nutrient absorption and intestinal mucosal barrier maintenance [16]. Recent studies revealed that gut microbiota was a vital barrier for host against the invasion and colonization of pathogenic bacteria, implying its crucial roles in the prevention and amelioration of diseases [17, 18]. However, the ruminant gut microbiota was dynamically varied and influenced by multiple factors such as feed, animal species and external environment [19, 20]. Currently, the significance of milk replacer has been widely acknowledged as a result of its role in growth performance, immunity and health maintenance, but few reports have been published on the effect of milk replacer on gut microbiota of YBGs [21]. The present study investigated the effect of milk replacer administration on gut microbiota of YGBs and characterized the gut microbial shifts during the early period after birth.

Given feces cannot fully reflect the composition and diversity of gut microbial community, we selected the intestinal contents as the research object [22]. Age is an important factor affecting the structure and composition of gut microbial community [23]. Several studies have revealed that mammalian gut microbiota was normally affected by species, genotype and diet during development and reached stability at maturity [24, 25]. Wang et al. found that the differences of gut microbial diversity between juvenile and adult Boer goats were not significant [26]. Similarly, we observed that the alpha diversity of gut bacterial community did not change significantly with the age of YBGs. However, recent studies have revealed a dramatically increased alpha diversity of gut microbiota in musk deer, cattle and piglet with age, which was inconsistent with our observation in YBGs [27, 28]. We speculated that there may be differences in the composition and development of gut microbiota between different species and the gut microbiota of YBGs may reach a stable state at an earlier age. Remarkably, although microbial diversity was not dramatically different between different age groups, the proportion of some bacterial genera altered. We observed that the number and types of beneficial and functional bacteria increase with age, which was beneficial to realize the functional diversity of the gut. Moreover, some beneficial bacteria such as *Lachnospiraceae*, *Ruminococcaceae*, *Ruminiclostridium*, *Eubacterium* and *Barnesiella* significantly decreased and subsequently increase with age. This may be the result of the evolution of host towards a better structure during development.

Increasing evidence revealed the close relationship between gut microbial alterations and diet [29, 30]. Ruminant gut microbiota in infancy is more sensitive to dietary changes due to the immature gastrointestinal tract. Hu et al. found that the alpha diversity of piglets was apparently decreased during the early period after weaning due to the sudden diet transition from breast milk to solid feed [28]. Our previous research has demonstrated that milk replacer administration could not change the diversity of rumen microbiota of early-weaned YBGs, but the composition of the rumen microbiota has changed [14]. In the current study, we noticed that the milk replacer administration had no effects on the structure and diversity of the gut bacterial community of YBGs, which may be the results of the similarity in composition and physical structure of milk replacer and breast milk without causing stress response. Interestingly, although milk replacer administration and changing age did not alter the microbial diversity of YBGs, the percentage of some intestinal bacteria changed. At the phylum level, the ratio of *Firmicutes* in the gut microbiota of milk replacer-treated YBGs increased, whereas the ratio of *Cyanobacteria*, *Actinobacteria* and *Proteobacteria* decreased as compared to control YBGs. Remarkably, our previous study also indicated that milk replacer supplementation significantly reduced the abundance of *Actinobacteria* in the rumen of YBGs [14]. The *Firmicutes* is mainly responsible for the digestion of cellulose and its higher abundance in the intestinal environment contributes to meet the nutrition and energy requirements of animals during growth and development [31]. Moreover, *Firmicutes* contains large amount of gram-positive bacteria and some of them are regarded as beneficial bacteria, which contribute to against pathogenic invasion and maintain intestinal microflora balance [32]. Most members of *Cyanobacteria* can produce toxic cyanotoxin, which seriously threaten the health of human and animal [33]. It has been demonstrated that the relative abundance of *Actinobacteria* in the gut of diarrheal goat was dramatically increased [26]. In addition, the synergy of *Actinobacteria* with one partner or host can easily be transformed into a pathogenic interaction with another [34]. *Proteobacteria* mainly consists of many gram-negative bacteria such as *Vibrio cholerae*, *Helicobacter pylori*, *Salmonella* and *Escherichia coli*, which could result in diarrhea, gastritis, vomiting, gastrointestinal ulcers and even death, posing a great threat to animal health [35, 36].

Previous research has demonstrated that milk replacer supplementation ameliorated the gut microbiota of early-weaned yak and increased the abundance of bacteria involved in the utilization of fibrous and non-fibrous carbohydrates [37]. Consistent with previous study, this study also indicated that milk replacer administration improved the gut microbial composition of early-weaned YBGs, characterized by an increased abundance of some potentially beneficial gut bacteria and carbohydrate-degrading bacteria. Specifically, the proportion of *Lachnospiraceae*, *Bifidobacterium*, *Prevotellaceae*, *Roseburia* and *Akkermansia* in gut microbiota of milk replacer-treated YBGs increased, whereas the ratio of *Porphyromonas*, *Brevundimonas*, *Flavobacterium*, *Stenotrophomonas*, *Propionibacterium*, *Acinetobacter*, *Enterococcus* and *Clostridium* decreased. *Lachnospiraceae* was considered as potential probiotic in the rumen and intestine, displaying a negative correlation with intestinal inflammation [38]. *Bifidobacterium*, an important intestinal beneficial bacterium, displays multiple important physiological functions, such as anti-tumor, anti-aging and improving gastrointestinal function and immunity [39]. Importantly, *Bifidobacterium* can also improve the intestinal environment and inhibit the proliferation of pathogenic bacteria through producing antimicrobial peptides and organic acids [40]. *Akkermansia* was conducive to maintain gastrointestinal health and metabolic balance and decrease the risk of diabetes, obesity and inflammation [41]. Furthermore, *Akkermansia* was also involved in the improvement of gut barrier function and the regulation of immune homeostasis in the gut mucosa [42]. *Prevotellaceae* and *Prevotella* of the gut can degrade polysaccharide and high carbohydrate [43]. Both *Porphyromonas* and *Brevundimonas* were pathogenic bacteria, which can accelerate atherosclerosis and cause bacteremia, respectively [44, 45]. *Flavobacterium*, an opportunistic pathogen, can cause pneumonia, meningitis and sepsis as the host immunity decreases [46]. *Stenotrophomonas*, an emerging pathogens, was closely related to bacteremia, while *Propionibacterium* can cause Endocarditis, meningitis and dermatopathy [47, 48]. *Acinetobacter*, mainly inhabiting in the gastrointestinal tract, respiratory tract, skin and genitourinary tract, was opportunistic pathogen, which can result in pneumonia, endocarditis, bacteremia, as well as urinary and skin infections [49, 50]. *Enterococcus* has been demonstrated to cause life-threatening sepsis, cardio-periostitis and meningitis [51]. Additionally, many antibiotics commonly used in the clinic failed in the treatment of *Enterococcus* infection, due to the inherent and acquired resistance [52]. This study conveyed a crucial message that milk replacer administration gradually ameliorated the gut microbial composition via increasing the proportion of beneficial and pathogenic bacteria. *Clostridium* was previously reported to play an important role in causing necrotizing enterocolitis [53]. *Clostridium* has also been shown to be closely associated with intestinal toxemia and diarrhea in ruminants and its toxins affects the host health via multiple pathways [54]. Furthermore, we also observed that some potential beneficial bacteria in jejunum (*Ruminococcus* and *Ruminococcaceae*) and cecum (*Ruminococcaceae*, *Christensenellaceae* and *Ruminiclostridium*) of the milk replacer-treated YBGs significantly decreased and subsequently increased. *Ruminococcaceae* displayed the ability to degrade cellulose and starch and was closely related to feed efficiency in lamb and cattle [55]. Moreover, *Ruminococcaceae* has long been thought to be potential beneficial bacterium because of the positive regulation of the immune system and intestinal environment [56]. Remarkably, recent studies demonstrated that the abundance of *Ruminococcaceae* in the gut microbiota was negatively correlated with liver cirrhosis, non-alcoholic fatty liver and increased intestinal permeability [57]. *Ruminiclostridium*, as a potential beneficial bacterium in the gut, not only involved in the positive regulation of the growth performance but also could secrete short-chain fatty acids, which was conducive to maintain functionality and morphology of intestinal epithelial cells and the regulation of intestinal microbiota balance [58]. Remarkably, some of these bacteria, such as *Ruminococcus*, *Ruminococcaceae*, *Akkermansia*, *Eubacterium*, *Pseudobutyrivibrio*, *Roseburia* and *Butyricimonas* can also produce short-chain fatty acids (SCFAs), which could regulate intestinal permeability and maintain normal physiological functioning of the gut [59]. Moreover, SCFAs can also regulate energy intake through the brain-gut axis to alleviate the development of obesity and diabetes [60, 61].

## Conclusion

Taken together, this study indicated the gut microbial alterations in YBGs with the effect of age and milk replacer. Our results indicated that the difference in microbial diversity in YBGs with different ages was not significant, but the types and proportion of some beneficial bacteria on days 25, 45 and 75 were higher than that on day 15, which was conducive to the stability of the intestinal environment and realize the intestinal functional diversity. Moreover, milk replacer administration could improve the gut microbial composition and structure by increasing proportion of beneficial and harmful bacteria. These findings enriched the knowledge of gut microbiota in YBGs. Importantly, this study also expanded our understanding of the potential benefits of milk replacer and convey an important message that milk replacer may serve as a good applicant for ameliorating gut microbiota in early-weaned YBGs. However, several limitations presenting in this study need to be noticed, such as small sample size, dietary habit and individual variation.

## Data Availability

Yes.
